# The rhizosphere microbiota of plant invaders: an overview of recent advances in the microbiomics of invasive plants

**DOI:** 10.3389/fmicb.2014.00368

**Published:** 2014-07-23

**Authors:** Vanessa C. Coats, Mary E. Rumpho

**Affiliations:** ^1^Department of Molecular and Biomedical Sciences, University of MaineOrono, ME, USA; ^2^Department of Molecular and Cell Biology, University of ConnecticutStorrs, CT, USA

**Keywords:** rhizosphere, microbiome, plant–microbe interactions, invasive plant, soil

## Abstract

Plants in terrestrial systems have evolved in direct association with microbes functioning as both agonists and antagonists of plant fitness and adaptability. As such, investigations that segregate plants and microbes provide only a limited scope of the biotic interactions that dictate plant community structure and composition in natural systems. Invasive plants provide an excellent working model to compare and contrast the effects of microbial communities associated with natural plant populations on plant fitness, adaptation, and fecundity. The last decade of DNA sequencing technology advancements opened the door to microbial community analysis, which has led to an increased awareness of the importance of an organism’s microbiome and the disease states associated with microbiome shifts. Employing microbiome analysis to study the symbiotic networks associated with invasive plants will help us to understand what microorganisms contribute to plant fitness in natural systems, how different soil microbial communities impact plant fitness and adaptability, specificity of host–microbe interactions in natural plant populations, and the selective pressures that dictate the structure of above-ground and below-ground biotic communities. This review discusses recent advances in invasive plant biology that have resulted from microbiome analyses as well as the microbial factors that direct plant fitness and adaptability in natural systems.

## INTRODUCTION

Symbiotic relationships shaped the origin, organization, and evolution of all life on Earth. Originally defined as “the living together of unlike named organisms” (de Bary, 1878), the term symbiosis has traditionally been applied to associations like mutualism, commensalism, and even parasitism (Parniske, 2008). More recent symbiosis research is expanding this definition to encompass a role of microbial symbiotic relationships in far-reaching themes of biology such as speciation, evolution, and coadaptation (Margulis, 1993; Klepzig et al., 2009; Carrapiço, 2010; Lankau, 2012). The association and close relationships of organisms that cohabitate are vital for the growth and development of all eukaryotic organisms (Carrapiço, 2010; McFall-Ngai et al., 2013). These associations (=symbiotic networks of microorganisms) shape natural landscapes and directly influence the evolutionary trajectory of individual species and entire ecosystems (Gilbert, 2002; Klepzig et al., 2009).

Plant invasions are a global concern because they pose a direct threat to biodiversity and natural resource management, especially in protected areas (i.e., public lands, refuges, conservations, etc.; Foxcroft et al., 2013). For a plant to be considered invasive (and not just naturalized) it must be non-native to the ecosystem in question and it must cause environmental damage (i.e., detrimental effects on native flora and fauna) or harm humans (Invasive Species Advisory Committee [ISAC], 2006). Invasive plant science represents a crossroads of diverse opinions derived from many economic, ecological and societal interest groups, and this has lead to disputes regarding the correct approach to invasive plant issues (Simberloff et al., 2013). To further complicate the issue, plant classification as “invasive” or “weedy” is often based more on human perceptions and opinions than on actual data regarding the economic, societal, or environmental impact of the plant taxon (Hayes and Barry, 2008). However, the environmental consensus supports severe ecological damage by plants deemed invasive in protected areas and significant reductions in the biodiversity of native species resulting from plant invasions. Comprehensive reviews of invasive plant impacts have covered the ecological effects of invaders (Pyšek et al., 2012), nutrient cycling modifications (Ehrenfeld, 2003; Liao et al., 2007), mechanisms of plant invasion (Levine et al., 2003), hybridization, and competition (Vila et al., 2004). Synthesizing accurate predictions of the invasive potential of specific plant taxa has proven difficult and there is no universal trait that can be collectively applied to predict invasiveness (Rejmanek and Richardson, 1996; Richardson and Pysek, 2006; Hayes and Barry, 2008; Thompson and Davis, 2011; Morin et al., 2013). A standard approach is needed for accurate impact assessment and the development of a new global database suitable to make future predictions of problem taxa (Morin et al., 2013).

The rhizosphere microbiome comprises the greatest diversity of microorganisms directly interacting with a given plant; therefore, it has a tremendous capacity to impact plant fitness and adaptation. Bacterial and fungal communities in the rhizosphere affect plant immunity (van Wees et al., 2008; Ronald and Shirasu, 2012), pathogen abundance (Berendsen et al., 2012), nutrient acquisition (Jones et al., 2009; Richardson et al., 2009), and stress tolerance (Doubkova et al., 2012; Marasco et al., 2012). Traditional hypotheses for plant invasion, such as enemy release hypothesis (ERH; Klironomonos, 2002; Mitchell and Power, 2003; Blumenthal, 2006; Liu and Stiling, 2006; Reinhart and Callaway, 2006; Blumenthal et al., 2009; Eschtruth and Battles, 2009), accumulation of local pathogens (ALP; Eppinga et al., 2006), enhanced mutualist hypothesis (EMH; Marler et al., 1999; Reinhart and Callaway, 2004; Parker et al., 2006), and plant–soil feedbacks (Ehrenfeld, 2003; Ehrenfeld et al., 2005; Bever et al., 2012), all point directly to the rhizosphere microbiome, in its entirety, as the primary mediator of plant establishment and success.

The study of soil microbial communities once relied on laboratory culture techniques, phospholipid fatty acid analysis (PFLA), denaturing gel gradient electrophoresis (DGGE), and terminal restriction fragment length polymorphism (TRFLP; Zhang and Xu, 2008; van Elsas and Boersma, 2011). Early on, culture-based approaches revealed “the great plate count anomaly” wherein only about 1% of visible microscopic cells can be cultured using conventional techniques (Staley and Konopka, 1985; Zhang and Xu, 2008; Stein and Nicol, 2011). The DNA technologies available today use genetic information to model the structure and composition of a microbial community (Venter et al., 2004; Tringe and Rubin, 2005; Hugenholtz and Tyson, 2008; Kunin et al., 2008; Vakhlu et al., 2008; Marguerat and Bähler, 2009; Metzker, 2010; Wooley et al., 2010; Simon and Daniel, 2011; Sun et al., 2011; van Elsas and Boersma, 2011; Thomas et al., 2012; Yousuf et al., 2012; Bibby, 2013; Mathieu et al., 2013). Capable of generating millions of base pairs in a matter of hours for only a few thousand dollars, the primary limitation to next-gen sequencing technologies is handling the expansive datasets and applying appropriate statistical analyses to address the biological questions at hand (Metzker, 2010).

The link between the rhizosphere microbial community and invasive plant success has been studied for many years (Van der Putten et al., 2007; Pringle et al., 2009; Berendsen et al., 2012; Bakker et al., 2013). Invasive plants provide a unique perspective to study the effects of the rhizosphere microbiome on plant fitness, the role evolutionary interactions play in structuring the plant ecology observed at present, and the potential for directed control and management of invasive plants. The aim of this review was to focus on recent insights into plant–microbe interactions in the rhizosphere of invasive plants. We were interested in studies that used a sequencing based approach to investigate the rhizosphere microbiome of invasive plants. Surprisingly, we found that few invasive plant scientists have moved beyond traditional methods of soil community analysis (i.e., DGGE) regardless of the increasing availability of next-gen sequencing platforms. We discuss the current microbiome data for invasive plants with regard to popular mechanisms of plant invasion (i.e., enemy release, novel symbiont, etc.). Particular attention has been given to rhizosphere microbiome analysis and what this methodology reveals about microbial symbiotic networks in the soil as contributing factors to the development and progression of plant invasions in terrestrial ecosystems.

## RHIZOSPHERE MICROBIOTA ARE A KEY COMPONENT OF PLANT FITNESS

Over 400 million years ago, during the Paleozoic era, the evolution of land plants was made possible by a symbiosis between mycorrhizal fungi and the common ancestor of land plants (Wang and Qiu, 2006; Humphreys et al., 2010). This association resulted in a fitness advantage and enhanced stress tolerance that was critical for the establishment of terrestrial plants (i.e., increased access to water and mineral nutrients). Evidence of microbial symbiosis is apparent in the oldest lineages of land plants, the liverworts. The arbuscular mycorrhizal (AM) symbioses of liverworts significantly promote photosynthetic C uptake, acquisition of P and N from the soil, growth, and asexual reproduction (Humphreys et al., 2010). Mycorrhizal symbioses undoubtedly demonstrate the importance of symbiotic relationships in terrestrial ecosystems and have been credited for stimulating the diversification of both plant hosts and fungal symbionts (Wang and Qiu, 2006).

**FIGURE 1:**
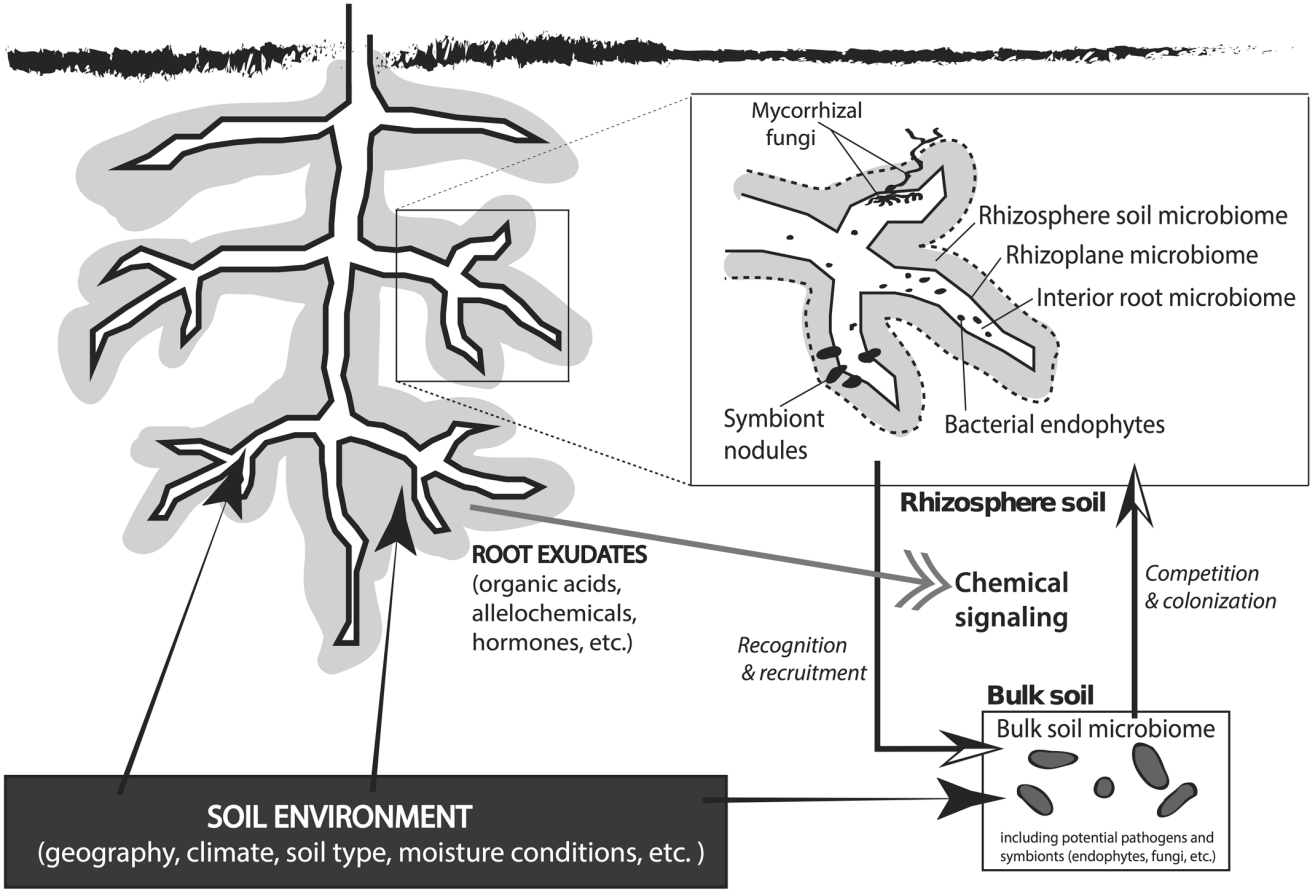
**An overview of plant–microbe interactions that occur in rhizosphere and bulk soils beneath a plant.** The soil environment has a direct effect on the plant, the rhizosphere microclimate, and the microbial community in the bulk soil. Root exudates from the plant direct chemical signaling between the plant and the microbial symbiotic network in the soil matrix. Rhizobiota recognize root exudate signals and are recruited to the rhizoplane or root interior. Bulk soil microbes compete for space to colonize the rhizosphere, which results in a rhizosphere microbial community that is derived from the total microbial population in the bulk soil. The microenvironment in the rhizosphere includes the rhizosphere microbiome (<3–5 mm of the root), rhizoplane microbiome (at root–soil interface), and the interior root microbiome. Common symbiotic interactions in the root zone include mycorrhizal fungi, bacterial endophytes, and symbiont nodules.

The soil microbial community constitutes a major portion of a plant’s symbiotic network. Soil is the greatest reservoir of microbes that affect plant growth, fitness, fecundity, and stress tolerance (reviewed by Buée et al., 2009; Faure et al., 2009; Lambers et al., 2009; Lugtenberg and Kamilova, 2009; Chaparro et al., 2012; Doornbos et al., 2012; Bakker et al., 2013). All plants maintain a direct interaction with soil microbes in the rhizosphere, which is the soil compartment immediately surrounding the root wherein plant root exudates directly influence the structure and function of the soil microbial community (**Figure 1**; Hiltner, 1904; Hartmann et al., 2008). The sugars, amino acids, flavonoids, proteins, and fatty acids secreted by plant roots help to structure the associated soil microbiome (Badri et al., 2009; Dennis et al., 2010; Doornbos et al., 2012) and these exudates vary among plant species and between genotypes (Rovira, 1969; Micallef et al., 2009). The quantity and composition of root exudate fluctuates with plant developmental stage and the proximity to neighboring species (Chaparro et al., 2012). Microbes growing in the nutrient rich rhizosphere produce molecular signals that promote plant fitness and growth (i.e., hormones) and can disrupt inter-plant communication in natural systems (Faure et al., 2009; Sanon et al., 2009).

Microbes in the rhizosphere can provide a direct access to limiting nutrients (e.g., N_2_ fixing symbiont) or increase the total surface area of the root system (e.g., mycorrhizal fungi). Many reviews have already covered the positive effects of beneficial root symbionts in the rhizosphere (Buée et al., 2009; Bakker et al., 2013), factors affecting rhizosphere microbial communities (Philippot et al., 2013), and the microbial effects on plant health (Berendsen et al., 2012; Berlec, 2012; Bever et al., 2012) and stress tolerance (Rodriguez et al., 2008).

Antagonistic interactions derived from microbial pathogens play critical roles in determining the genetic structure and spatiotemporal abundance of a plant (Gilbert, 2002; Blumenthal et al., 2009). Pathogenic microbes impose selective pressures on a plant population that favor a specific genetic structure within the host plant community and this stimulates evolutionary change over time (Gilbert, 2002). In natural systems, pathogens mediate plant competition and affect spatiotemporal distribution of individuals within the plant community by creating inhabitable and uninhabitable areas within the ecosystem (Gilbert, 2002). The Janzen-Connell hypothesis postulated that pathogen and host densities are responsible for the observed distribution of a plant species by affecting the establishment success of seedlings (Packer and Clay, 2000). A high density of *Pythium* sp. in the soil beneath parental *Prunus serotina* trees was observed to prohibit the establishment of seedlings in the immediate vicinity (0–5 m), but not seedlings growing at greater distances (25–30 m; Packer and Clay, 2000). Thus, pathogen accumulation beneath parent plants functions to promote seedling distribution and reduce competition between the parent plant and its offspring.

## INVASIVE PLANTS DISRUPT NATIVE SYMBIOTIC NETWORKS

The introduction of non-native plants can disrupt native symbiotic networks in the soil and change local grazing patterns for insects and fauna (Elias et al., 2006; Klepzig et al., 2009). Introduced plants alter patterns of nutrient cycling (Laungani and Knops, 2009) and cause chemical changes in the soil environment (i.e., allelopathy; Cipollini et al., 2012). Often these non-native invaders bring novel traits to the environment that put native plants at a disadvantage (Van der Putten et al., 2007; Laungani and Knops, 2009; Perkins et al., 2011). Plant–microbe interactions may assist invasive plants with outcompeting native flora using mechanisms that include allelopathy-mediated suppression of native rhizosphere microbes and beneficial symbionts (Stinson et al., 2006; Callaway et al., 2008), the accumulation of native plant pathogens in the invaded soils (Mangla et al., 2008), and changes in nutrient cycling dynamics that favor the exotic plant (Ehrenfeld et al., 2001; Ehrenfeld, 2003; Laungani and Knops, 2009). Increased availability or access to vital nutrients provides a competitive advantage to invasive plants and facilitates significant biomass accumulation (Blumenthal, 2006; Blumenthal et al., 2009).

Allelopathic plants are among the most aggressive invaders of non-native ecosystems because non-native plants with the ability to synthesize toxic chemicals are often at a competitive advantage (Lankau, 2012). *Allaria petiolata* (garlic mustard) produces allelopathic chemicals that target beneficial microbes like AM symbionts of native plants (Stinson et al., 2006; Callaway and Vivanco, 2007; Callaway et al., 2008). *A. petiolata* also demonstrated an increased production of toxic chemicals when growing in non-native regions that contain a greater competitive interspecific density, implicating the allelopathic effects as the primary invasive characteristic (Lankau, 2012). The introduction of novel allelochemicals into an environment affects the structure of the soil microbial community and the microbial biodiversity, especially if these chemicals have antimicrobial activity or function as metal chelators (Inderjit et al., 2011). Soil microbes are the first line of defense toward novel chemicals in a native ecosystem. They mediate much of the allelopathic effect in ways as simple as the ability to degrade or detoxify compounds before they accumulate in the soil and inhibit native plant growth (Cipollini et al., 2012).

Invasive plants outcompete native plants by accumulating large concentrations of native plant pathogens in the soil (Eppinga et al., 2006; Mangla et al., 2008). A release from microbial pathogens, insect pests, and herbivores of the native range is one mechanism behind the success of invasive plants (Klironomonos, 2002; Mitchell and Power, 2003; Reinhart and Callaway, 2006; Blumenthal et al., 2009), but the distribution of pathogens in the invasive range is just as important for defining competition with native flora. Root exudates of *Chromolaena* *odorata*, a severely destructive tropical weed, concentrate *Fusarium* sp. spores to a level 25-times greater than that observed in the root zone of native plants (Mangla et al., 2008). Thus, these plants exacerbate and exploit the native biotic interactions and gain a competitive advantage.

Many, but not all, invasive plants alter patterns of nutrient cycling in the invasive range (Perkins et al., 2011). Changes in the N cycling dynamics in the soil are a frequent consequence of invasive plant introduction (Ehrenfeld, 2003; Mack and D’Antonio, 2003; Laungani and Knops, 2009; Perkins et al., 2011). Non-native species can change the quality and quantity of leaf litter (Ehrenfeld et al., 2001), modify local decomposition rates (Kourtev et al., 2002a; Elgersma et al., 2012), and disrupt local feedback mechanisms in the soil system (Ehrenfeld et al., 2005). For example, *Pinus strobus* is an invader of N-poor grasslands that demonstrates a higher N residence time in the plant tissues than native species (Laungani and Knops, 2009). This increased residence time facilitates the accumulation of twice as much N in plant tissues and up to four times as much N in the photosynthetic tissues, relative to native grasses (Laungani and Knops, 2009). The differences in N utilization between non-native and native plants create a positive feedback in the soil that significantly increases N availability and results in increased total C gains, both of which allow *P. strobus* to gain a competitive advantage (Laungani and Knops, 2009).

## MICROBIAL IMPACTS ON PLANT ESTABLISHMENT AND PROLIFERATION

Not all microbes are found ubiquitously throughout soils around the world, and thus, soil microbes are not exempt from fundamental evolutionary processes of geographic isolation and natural selection (Rout and Callaway, 2012). Plant–microbe interactions in the rhizosphere (beneficial, pathogen, etc.) can dictate whether the plant is capable of naturalization and the possibility of an invasive growth habit. Pringle et al. (2009) proposed three criteria to model how mycorrhizal symbioses influence the outcome of a plant invasion: (1) the type of plant–fungi relationship (obligate or facultative) from the plant perspective; (2) if the relationship was specific or flexible, meaning the plant associates with one mycorrhizal fungus versus many; and (3) whether these microbial symbionts were found in the introduced range (Pringle et al., 2009). According to this model, obligate symbionts prevent the growth of non-native plants if the microbial symbiont is not already present in the introduced region, nor is it co-introduced with the host plant. Facultative symbioses are often less restrictive because the plants may form novel beneficial symbioses with suitable replacement microbes in the non-native range, or survive without the symbiont. Consequently, the symbiotic flexibility in facultative symbioses enhances the likelihood of favorable plant adaptations and the development of invasive populations in the introduced region (Pringle et al., 2009).

In the introduced region, the soil microbial community mediates plant abundance and disturbance of the soil can influence the progression of a plant invasion. A removal of the above-ground plant community coupled with little or no physical disruption of the soil is classified as Type I soil disturbance. A Type II soil disturbance includes physical disruption of the soil matrix in addition to removal of the above-ground plant biomass (Fukano et al., 2013). Type I disturbances leave the soil microbial community intact, whereas Type II disturbances completely disrupt the structure of the microbial community. Interestingly, the growth of non-native species is enhanced when they are rare in the ecosystems subjected to Type I disturbance (Fukano et al., 2013). In contrast, type II disturbances give native species an advantage and require non-native invaders to maintain a higher competitive ability. Thus, a physical disturbance that alters the composition of the soil microbial community favors native plants, yet the opposite result occurs (enhanced fitness of non-native plants) if the soil microbial community remains intact.

## THE RHIZOSPHERE MICROBIOTA OF INVASIVE PLANTS

**FIGURE 2:**
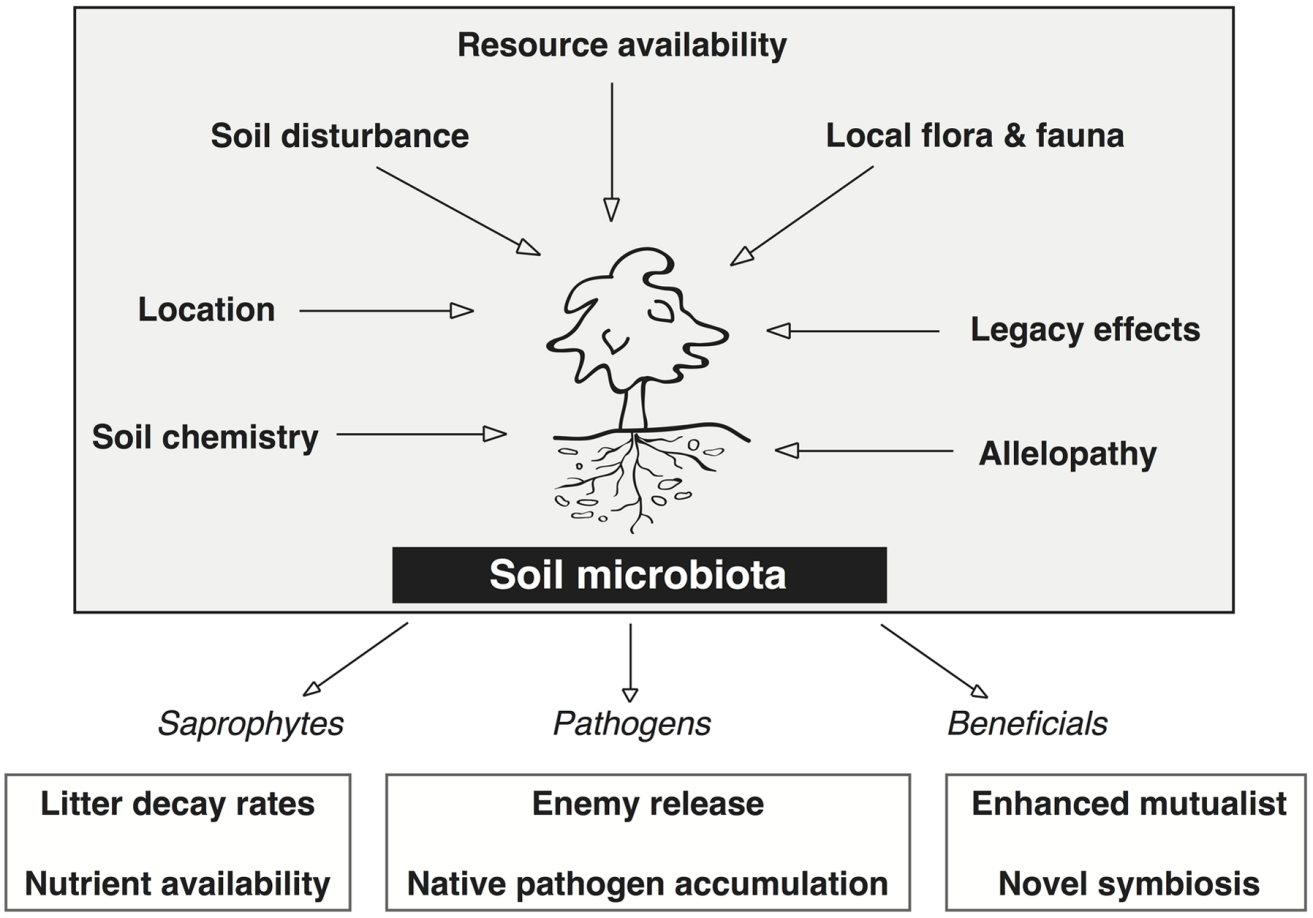
**Factors that directly affect the soil microbiota associated with invasive plants and the positive feedbacks on the plant invasion derived from each major group (saprophytes, pathogens, and beneficials).** Each of the seven factors directly affect the microbial community structure and function in the soil by imposing some degree of selective pressure wherein certain microbes are not capable of surviving. These seven factors dictate the relative abundance of saprophytes, pathogens, and beneficials that are able to associate with the plant. Two mechanisms of plant invasion that lead to positive feedbacks from these plant–microbe interactions are shown for each group of soil microbes.

The rhizosphere microbiota of non-cultivated plant systems provide a better platform to study the critical plant–microbe interactions that affect plant fitness and adaptability because they are under less anthropogenic control than agricultural systems (Philippot et al., 2013). **Figure 2** depicts seven biotic and abiotic factors that together determine the presence or absence of specific microbiota in the soil microbiome of natural systems. Factors such as soil disturbance, local flora and fauna, and allelopathic effects from the plant each impose a selective pressure on the soil microbial community. The cumulative effect of these selective pressures is what determines the frequency and abundance of microbes in the soil, and thus, what microbes the plant is able to recruit into the rhizosphere.

Microbiome analysis of rhizosphere microbiota associated with invasive *Berberis thunbergii* in Maine showed that environmental factors alone cannot explain the structure of the rhizosphere microbial community associated with this plant in the invasive range. Coats et al. (2014) used amplicon pyrosequencing to assess effects of environmental factors on the bacterial and fungal communities in the rhizosphere of *B. thunbergii* (Japanese barberry) from invasive stands in coastal Maine, USA. The effects of soil chemistry, location, and surrounding plant canopy cover were investigated and a high degree of spatial variation in the rhizosphere microbial communities of *B. thunbergii* was reported. Bulk soil chemistry had more of an effect on the bacterial community structure than the fungal community. An effect of location was detected in the rhizosphere microbial community, but it was less significant than the effect of surrounding plant canopy cover. The significant effects of these environmental factors on the structure of the rhizosphere microbial community associated with *B. thunbergii* suggests some soils and/or plant communities are more prone to plant invasions based on the soil microbial communities they foster.

The microbial diversity in the rhizosphere includes many species of bacteria, archaea, fungi, oomycetes, viruses, and various microfauna (nematodes, protozoa, etc.; reviewed by Buée et al., 2009; Bever et al., 2012; Philippot et al., 2013). The rhizosphere microbiome differs from the bulk soil and between plant species. Using a metatranscriptomic approach, Turner et al. (2013) identified kingdom level differences in the rhizosphere bacterial communities of wheat, oat, and pea plants. The fungal diversity in the rhizosphere also varied significantly between these crop plants. Investigations that have focused on the interactive effects between major microbial groups in the rhizosphere have revealed a joint effect of fungal endophytes and AM fungi that promotes plant growth (Larimer et al., 2010). Bacterial endophytes have been observed to enhance competition by invasive plants through providing the plant with increased access to nutrients (Fe and P) and by producing plant growth promoting hormones (IAA; Rout et al., 2013). When comparing native and non-native plants with DGGE, Xiao et al. (2014) found that the soil fungal communities were more affected by the invasive plant than the native plant and the modifications to the fungal community promoted invasive plant growth. Differences in the rhizosphere pathogen communities of related *Phragmites australis* haplotype populations (a native and non-native) have also demonstrated that non-native species cultivate different soil pathogen communities than native plants regardless of the genetic similarity of the host plant (Nelson and Karp, 2013).

## RHIZOSPHERE MICROBIOME IN NATIVE AND INVASIVE RANGE SOILS

Recent investigations that have contrasted plant–microbe interactions in the native and invasive range have focused on the net effect of soil biota on plant growth, plant allelopathic responses, and the rhizosphere microbiome. The rhizosphere microbiota (saprophytes, pathogens, and beneficials) each have positive effects on invasive plant growth (lower boxes of **Figure 2**). Stimulating saprophyte growth creates a positive feedback in the soil of invasive plants by increasing litter decay rates and nutrient availability (Van der Putten et al., 2007; Bever et al., 2012). The mutualistic associations and/or novel symbioses in the introduced range can enhance plant fitness by promoting plant growth, nutrient acquisition, and disease suppression (Van der Putten et al., 2007; Pringle et al., 2009; Berendsen et al., 2012; Bakker et al., 2013). The empirical evidence obtained from studies that compare plant–microbe interactions in each range support current microbe based theories of plant invasions and provide evidence for microbe enhanced plant fitness in the invasive range.

*Triadica sebifera* (Chinese tallow) is native in China and invasive in the US. Yang et al. (2013) studied the net effect of native and invasive range soil microbiota on the growth of *T. sebifera* and four co-occurring plant genera (*Liquidambar*, *Ulmus*, *Celtis*, and *Platanus*). Native range soils had no effect, or a negative effect, on *T. sebifera* performance yet there was always a positive effect of invasive range soil on plant survival and biomass production. A greater biomass was observed for the invasive plants grown in active soil mix than in sterilized or fungicide-treated soils. Higher mycorrhizal colonization of *T. sebifera* was found on plants growing in the invasive range soil. Interestingly, there was no effect of native or invasive range soil on the other four genera examined, and native plants maintained higher mycorrhizal colonization rates in native soil than invasive range soil. These results not only support Enhanced Mutualist and Pathogen Release Hypotheses, they also indicate a significant specificity in the plant–microbe interactions for some plant species that contribute to invasive plant growth.

The allelopathic response of invasive plants can differ between native and invasive ranges with greater allelopathic effects observed in the invasive range. Yuan et al. (2013) observed increased allelochemical content (total phenolics, total flavones, and total saponins) for *Solidago canadensis*, a native of the US that has developed invasive populations in China. The increased production of allelopathic chemicals by *S. canadensis* in the invasive range also coincided with a greater inhibition of native plant seedlings. Whether the increase in allelochemical production is solely a result of the plant–microbe interactions remains unclear, although it would seem to be a beneficial plant response to the development of novel interactions with foreign soil microbiota.

The most comprehensive investigation of a rhizosphere microbiome associated with an invasive plant was conducted on *B. thunbergii*, a native of central Japan that is invasive in the US. The microbial community (Bacteria, Archaea, and Eukaryota) structure was modeled using amplicon pyrosequencing to compare rhizosphere communities of native *B. thunbergii* from central Japan (*n* = 8) with those from an invasive stand in the US (*n* = 5; Coats, 2013). A total of 432 genera were identified from all three domains in Japan and US rhizosphere soils combined, although only Eukaryotes from the lineage Fungi were included in this analysis. *B. thunbergii* rhizosphere soils from Japan and the US shared 171 genera, most of which were Proteobacteria (Bacteria) and Ascomycota (Fungi). Rhizosphere soil from Japan contained 71 unique genera and the US soils harbored 190 unique genera. A high degree of phylogenetic redundancy was observed within the microbial community at the phyla level, although the community structure was significantly different between samples from each region (Coats, 2013).

The apparent difference in the rhizosphere microbiota of *B. thunbergii* in native and invasive (non-native) soil supports our hypothesis that soil microbial communities are the primary mediators of invasive plant growth in non-native habitats. The data showed a significant effect of geographic location with less species diversity and increased abundance of pathogenic species observed in rhizosphere soils from the native range compared to the invasive range (Coats, 2013). Therefore, the microbial community shifts observed between the rhizosphere soil in the native and non-native ranges support Enemy Release and Enhanced Mutualist Hypotheses, as well as an increased access to nutrients via saprophyte stimulation and/or novel symbiont acquisition. Interestingly, Bacteria communities were more significantly different between rhizosphere samples from the two ranges than the Archaea or the Eukaryota communities (Coats, 2013).

Pathogen release, wherein exotic plants are not subjected to the heavy pathogen loads characteristic of native range soils in the non-native range, has been implicated as a common mechanism for plant invasions, especially when coupled with increased access to nutrients (Blumenthal, 2006; Blumenthal et al., 2009). The impacts of enemy release on a plant invasion are determined from two opposing factors: (1) plants’ “escape” from heavy pathogen loads in the native range and (2) the rate of accumulating pathogens in the introduced range (release = escape - accumulation; Mitchell and Power, 2003). Many genera that were found strictly in *B. thunbergii* rhizosphere soils from Japan are common plant pathogens, including *Clostridium*, *Enterobacter* (*Pantoea*), and *Serratia* (Schaad et al., 2001; Grimont and Grimont, 2006), and these putatively pathogenic microbes occurred in greater abundance in the native soils. For instance, two pathogenic *Serratia* species (*S. proteamaculans* and *S. marcescens*) constituted 1.8% of the total reads in some rhizosphere samples from Japan and as much as 52% of the total for other Japan rhizosphere samples (Grimont and Grimont, 2006; Coats, 2013). *Buttiauxella* was detected in every rhizosphere sample from Japan (compared to three US samples) and it comprised 8.5–70.1% of the total reads, although the average was approximately 30–35% per sample. *Stenotrophomonas*, another putative *Berberis* pathogen, comprised approximately 1–9% of the total reads in the native Japan soils but contributed very little (∼0.1% of the total reads) to the microbial community in the rhizosphere soil from the US (Coats, 2013).

The rhizosphere microbial communities associated with *B. thunbergii* also implicate a role for enhanced mutualism as one factor in the development of invasive populations (Coats, 2013). Some genera that are likely to be putative beneficial symbionts, such as *Glomus* (mycorrhizal fungi) and *Frankia* (N_2_-fixing actinomycete), were detected solely in rhizosphere communities of the invasive range. Other genera that also contain putative beneficials were detected in both regions, although their abundance was greater in the rhizosphere soil from the invasive range. Some of these genera are capable of symbiotic or free-living (diazotrophic) N fixation (e.g., *Bradyrhizobium*, *Rhizobium*, *Azospira*, etc.), whereas others are likely to function more like plant growth promoting rhizobacteria (e.g., *Bacillus* and *Pseudomonas*) that promote plant fitness by producing growth simulating phytohormones (Faure et al., 2009; Effmert et al., 2012), enhancing stress tolerance (Dimkpa et al., 2009; Kang et al., 2010; Pineda et al., 2010), or antagonizing pathogenic microbes that inhabit the root zone (Berendsen et al., 2012).

Alterations to N cycling dynamics are a commonly reported feature of *B. thunbergii* invasions in North American soils, which suggests saprophyte stimulation (via increased litter decay rates) and/or novel symbiont acquisition are responsible for the observed changes in the invasive range (Coats, 2013). Relative to native *Vaccinium* shrubs, *B. thunbergii* plants produce large quantities of N-rich biomass, N-rich leaf litter, and N-rich secondary metabolites (Ehrenfeld et al., 2001; Elgersma et al., 2012) and they harbor higher levels of extractable nitrate in the soil (Ehrenfeld, 1999). *B. thunbergii* preferentially uses nitrate (Ehrenfeld et al., 2001), a trait that facilitates out-competing ammonium utilizing plants (Gilliam, 2006), and these exotic plants have increased rates of nitrification in the soil rather than high N availability from mineralization (Kourtev et al., 2002b, 2003; Elgersma et al., 2011). The rhizosphere soil from *B. thunbergii* showed an increased abundance of nitrifying bacteria such as Nitrospirales (0.0–2.4%) and Nitrosomonadales (0.4–1.6%) in the invasive range soils relative to rhizosphere soils from the native range (0.0–0.3% and 0.0–0.2% for Nitrospirales and Nitrosomonadales, respectively; Coats, 2013). The data acquired by microbiome analysis show that differences in the microbial community structure between the two ranges corroborate previous investigations of soil N cycling beneath *B. thunbergii* in the invasive range. This metagenomic approach also identifies specific organisms that are likely to be the culprits behind changes in the N cycling patterns in the invasive range soil and that can be targeted during future investigation of the microbial function in the rhizosphere.

## FUTURE RESEARCH

Given the recent advances in high-throughput DNA sequencing and the availability of cost-effective microbiome analysis, it is time invasive plant biologists begin to focus on a full characterization of soil microbial communities in an effort to understand how changes or shifts in the rhizosphere microbiome are affecting the above-ground ecology. Metagenomics and metatranscriptomics provide a rapid means to investigate the genomics and gene expression that mediate plant–microbe interactions in the rhizosphere as well as provide much needed information regarding the metabolic capacity and ecological function of rhizosphere microbes. These plant–microbe interactions not only contribute to invasive plant growth and fitness, they also define the range of suitable habitats and areas of competitive advantage. Obtaining high quality predictions for the most susceptible habitats is the best way to prevent invasive plant introduction and subsequent damage. Microbiome profiling of soil, by programs such as the Earth Microbiome Project (http://www.earthmicrobiome.org/; Gilbert et al., 2010), will undoubtedly enhance prediction algorithms and help identify microbial components in regions of high or low susceptibility. However, the information gained from rhizosphere microbiome analysis is not limited to predictions and promoting a better understanding of plant–microbe interactions in natural ecosystems. Microbiome-based investigations will greatly assist in the development of microbial probiotics and/or targeted approaches to reclaiming habitats that have become heavily invaded (Berlec, 2012). Such an approach would continue to build on current methods of reducing cost and environmental damage caused by terrestrial invaders and focus efforts on prohibiting the initial establishment.

## CONCLUSION

The introduction and prevalence of invasive plants, and the threat of increasing invasion rates, substantiates the need to understand the mechanisms underlying the success of plants that become invasive. Symbiotic networks of microorganisms in the soil undoubtedly affect the naturalization of non-native plants in the introduced region and the ability of these plants to outcompete native species. Plant–microbe interactions in the rhizosphere directly contribute to plant fitness, nutrient acquisition, and stress tolerance. Therefore, the rhizosphere microbiome of a plant harbors a tremendous capacity to promote or inhibit invasive growth characteristics. Invasion mechanisms employed by some plants involve rhizosphere microbiome shifts between the native and invasive ranges. These microbial community shifts provide evidence in support of the Enemy Release and Enhanced Mutualist Hypotheses as well as corroborating plant–microbe feedbacks that lead to an enhanced resource acquisition beyond the limits of native flora.

## AUTHOR CONTRIBUTIONS

The manuscript was drafted by Vanessa C. Coats with editorial remarks from Mary E. Rumpho.

## Conflict of Interest Statement

The authors declare that the research was conducted in the absence of any commercial or financial relationships that could be construed as a potential conflict of interest.
